# AC Loss in High-Temperature Superconducting Bulks Subjected to Alternating and Rotating Magnetic Fields

**DOI:** 10.3390/ma16020633

**Published:** 2023-01-09

**Authors:** Wafa Ali Soomro, Youguang Guo, Haiyan Lu, Jianxun Jin, Boyang Shen, Jianguo Zhu

**Affiliations:** 1 Faculty of Engineering and IT, University of Technology Sydney, Sydney, NSW 2007, Australia; 2 School of Electrical and Information Engineering, Tianjin University, Tianjin 300072, China; 3 Electrical Engineering Division, University of Cambridge, Cambridge CB3 0FA, UK; 4 School of Electrical and Information Engineering, University of Sydney, Sydney, NSW 2006, Australia

**Keywords:** high-temperature superconductors, HTS bulks, rotating magnetic fields, AC loss

## Abstract

High-temperature superconductor (HTS) bulks have demonstrated extremely intriguing potential for industrial and commercial applications due to their capability to trap significantly larger magnetic fields than conventional permanent magnets. The magnetic field in electrical rotating machines is a combination of alternating and rotational fields. In contrast, all previous research on the characterization of electromagnetic properties of HTS have solely engrossed on the alternating AC magnetic fields and the associated AC loss. This research paper gives a thorough examination of the AC loss measurement under various conditions. The obtained results are compared to the finite element-based H-formulation. The AC loss is measured at various amplitudes of circular flux density patterns and compared with the AC loss under one-dimensional alternating flux density. The loss variation has also been studied at other frequencies. The findings in this research paper provide more insights into material characterization, which will be useful in the design of future large-scale HTS applications.

## 1. Introduction

Over thirty years have passed since the discovery of high-temperature superconductors (HTS). Since then, extensive research has focused on using the HTS material to create cutting-edge electric power appliances. With the help of superconducting technology, it is possible to develop small, light, and efficient electrical machines because HTS material enables a considerable reduction in size and an increase in machine efficiency [1,2]. There have been numerous applications of superconducting machines ranging from superconducting motors and generators to transformers [3]. Furthermore, novel technologies that are also made possible by superconductivity include superconducting fault current limiters [4] and superconducting magnetic energy storage [5]. HTS bulk magnets have also been a significant field of superconductivity study. As permanent magnets, they are capable of producing powerful magnetic fields. Unlike conventional permanent magnets, HTS bulks produced from RE-Ba-Cu-O (RE stands for rare-earth element) using a top-seeded melt growth process can trap significantly high magnetic fields at low temperatures. With increasing sample size and critical current density, the magnetic field intensity trapped in HTS permanent magnets may be significantly increased. HTS magnets now hold the world record for trapped magnetic field strength of 17.9 T [6]. This distinguishing feature makes trapped field magnets ideal aspirants for the development of compact and efficient electrical apparatuses having extraordinary power densities [7,8].

Moreover, the commercialization of the HTS is hindered by the dissipative interactions that take place when these materials are subjected to the alternating magnetic field. It is known as AC loss or power dissipative loss and is brought on due to the displacement of vortices within the material [3]. This power dissipation phenomenon might put an additional load on the cryocoolers. The geometric structure, the orientation of the magnetic field, and the dispersal of current density within the material’s domain are among a few of the variables that have a significant impact on this power dissipation. Therefore, it is crucial to study and characterize the electromagnetic behavior of HTS bulks to guarantee the practicability of commercial superconducting applications.

The HTS material only enters its superconducting state at the cryogenic temperature. Consequently, a cryogenic cooling unit, also known as a cryocooler, is placed when developing practical and commercial applications. This device removes the relevant heat in order to maintain a consistent temperature throughout the operation. The cryogenic system’s stability may be hampered by the AC loss, which has an impact on the device’s overall effectiveness. Therefore, it is very crucial to have a thorough grasp of the process and size of the AC loss in order to design and build new superconducting machines.

HTS trapped field magnets could be exposed to alternating and rotating magnetic fields when used in rotating machines. However, most electromagnetic studies in the literature concentrate on characterizing HTS material only under one-dimensional alternating magnetic fields. Numerous experimental methodologies published on AC loss, only emphasize on one-dimensional exposure of AC magnetic fields, due to the unavailability of reliable testing methods under rotating magnetic field conditions. Few studies have been published that provide some information on the magnetic movement [9,10] and the levitational force [11], but a comprehensive experimental study on the AC loss has not been presented. The electromagnetic characteristics of HTS materials in rotating magnetic fields need to be investigated in order to fully comprehend electromagnetic characteristics for the effective design of HTS rotating machines. Recent works by the authors [12,13,14] concentrate on numerical analyses of AC loss due to rotating magnetic fields, where it was discovered that such AC loss differs dramatically from one-dimensional magnetization when exposed to rotating magnetic fields. Although these numerical models are effective in determining how the HTS material will behave, experimental methods are equally crucial for examining the HTS electromagnetic characteristics.

The experimental methods for AC loss analysis described in earlier research, such as Electrical Method [15], Magnetic Method [16], and Calorimetric Method [17] are also carried out in the presence of one-dimensional (1D) alternating magnetic fields formed by the transporting current or by external excitations. In contrast, the magnetic field rotates in a two-dimensional pattern in applications such as three-phase transformers or electrical machines, where B and H may not be in the same direction. Because of the experimental restrictions in existing methods, it is not possible to evaluate the AC loss caused by exposure to rotating magnetic fields.

Recently, the authors have presented a unique experimental approach for measuring AC loss in rotating magnetic fields in [18], where only the experimental setup has been proposed. In this paper, a detailed investigation on the AC loss measurement has been presented using this novel experimental setup. A square sample of Melt-textured REBa2Cu3O7-x with RE2BaCuO5 excess HTS bulk sample is exposed to alternating and rotating magnetic fields with various amplitudes and frequencies. The HTS bulk is manufactured using seeded melt growth. The results are also compared with those from the numerical modeling. This detailed characterization may be beneficial for the further design of large-scale high-efficient superconducting rotating machines.

## 2. AC Loss Measurement Setup

**Figure 1:**
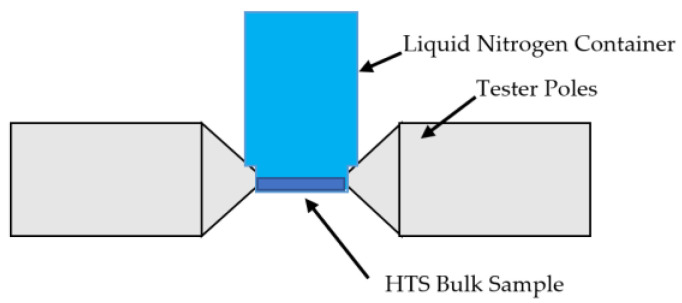
Tester arrangement incorporating the HTS bulk sample is placed inside the liquid nitrogen box.

**Figure 2:**
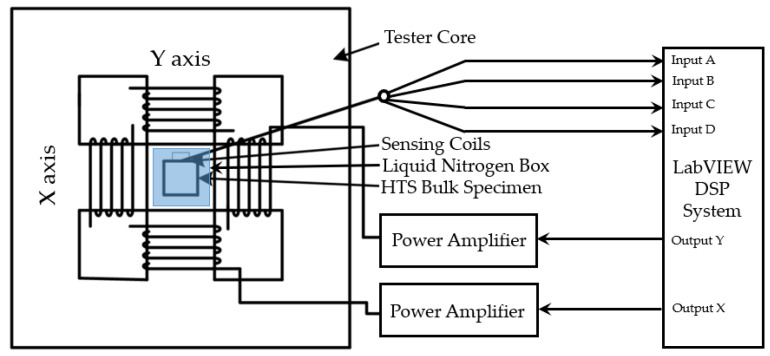
Block diagram of the SST and associated experimental setup.

In this study, a modified square specimen tester presented in [18] is utilized to study the AC loss behavior of HTS bulks under the rotating magnetic fields. The single sheet tester (SST) consists of vertically laminated, grain-oriented silicon steel sheet yokes. Due to the design of the yoke shapes, the tester has four wedge-shaped magnetic poles. There are two sets of excitation coils, which are connected in series to one another on the X- and Y-axes magnetic poles. Each coil encompasses 300 turns of enameled 1.6 mm copper wire. The HTS sample was kept immersed inside liquid nitrogen in a carefully constructed liquid nitrogen box that was placed in the center of the taster. The placement of the liquid nitrogen box is shown in Figure 1, along with the block layout of the 2D experimental setup as in Figure 2.

To produce the rotating magnetic field around the sample, two groups of exciting coils are excited. Using Labview and the power amplifiers, purely rotating magnetic fields are produced by excitation coils with a 90° phase difference. The tester’s rotating magnetic field induces magnetization in the test specimen which is measured along both axes by specifically fabricated and calibrated B- and H-sensing coils. An FPGA-based digital signal processing system is used for function generation and data collection as shown in Figure 2. The X and Y components of the magnetic field strength (Hx and Hy) and the flux density (Bx and By), respectively, are determined using specialized B and H coils. The B and H are determined by placing specialized sensing coils all around the specimen. The rotating AC loss is then calculated using the field-metric method by measuring B and H components. Furthermore, these measurements are made by inducing a voltage with a microvolt range in the coils.

One coil is put in the X-axis and the other in the Y-axis to measure the H components, while the B-sensing coils are wrapped across the middle of the specimen. To make the H-sensing coil for each axis, 100 turns of 0.06 mm enamel copper wire are wound around a 0.50 mm plastic former. The voltage induced in the coils is then employed in Equation (1) to calculate the H components on each axis [19]:

(1) $$H_{i}=\frac{1}{\mu_{0}K_{H_{i}}}\int V_{H_{i}}dt\;\;\;\;\;\;\left(i=x,y\right)\;\;\;\;\;\;$$

where $\mu_{0}$ is the permeability of air, $V_{H_{i}}$ is sensing coil voltage, and $K_{H_{i}}$ is the coil coefficient, which is obtained after the calibration of the coils [19].

Each coil for measuring the B components is made up of 20 turns of 0.1 mm enamel-insulated copper wire. The voltage induced in the coils is then employed in Equation (2) to calculate the B components on each axis:

(2) $$B_{i}=\frac{1}{K_{B_{i}}}\int V_{B_{i}}dt\left(i=x,y\right)$$

(3) $$K_{B_{i}}=N_{B_{i}}A_{sp}$$

where$\;V_{B_{i}}$ is the induced voltage in the B coils, $K_{B_{i}}$ is the coefficient of coil, which considers the cross-sectional area of the specimen and number of turns of the coil [19].

These observed instantaneous B and H values can provide information such as different loss characteristics, and the loci of H and B vectors. Additionally, power dissipation may also be determined once a rotating magnetic field is applied to the HTS sample using Poynting’s theorem [19]:

(4) $$P_{\left(2D\right)}=\frac{1}{T\rho_{m}}\int_{0}^{T}\left(H_{x}\cdot \frac{dB_{x}}{dt}+H_{y}\cdot \frac{dB_{y}}{dt}\right)dt\;\;$$

where *T* is the time period of one magnetization process, $\rho_{m}$ is the sample mass density, and *H_x_, H_y_* and *B_x_, B_y_* are the X and Y components of H and B, respectively.

Furthermore, one-dimensional AC magnetic fields can also be produced from the same testing system, by only exciting a certain set of coils in x- or y-axis as per testing requirements. In such case, the loss can be measured in a similar way using Poynting’s theorem [19], as in Equations (5) and (6):

(5) $$P_{\left(1Dx\right)}=\frac{1}{T\rho_{m}}\int_{0}^{T}\left(H_{x}\cdot \frac{dB_{x}}{dt}\right)dt\;\;$$

(6) $$P_{\left(1Dy\right)}=\frac{1}{T\rho_{m}}\int_{0}^{T}\left(H_{y}\cdot \frac{dB_{y}}{dt}\right)dt\;\;$$

## 3. Numerical Modeling Background

**Figure 3:**
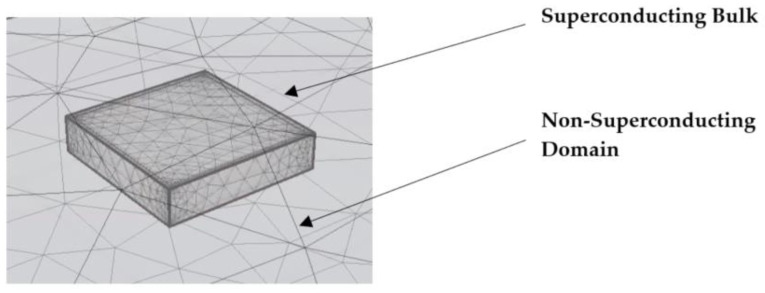
Meshed model of Superconducting Bulk sample.

Numerical modeling techniques are important to predict and discover all of the material characteristics without the necessity for time-consuming and costly experiments. We used a 3D H-formulation in this investigation, which is one of the highly studied finite element method (FEM) based numerical models. In terms of finite element modeling, three-dimensional (3D) models are favored over conventional two-dimensional (2D) models because a full 3D mesh considers a higher number of degrees of freedom, which provides more precise and accurate results. This model was created with COMSOL Multiphysics, a commercial FEA-based program which is widely used in superconducting research. A geometry similar to the specimen used in the experiment was considered in this model. It was assumed that the critical temperature is achieved across the sample, and hence, the thermal model was not considered to save the computation time. Figure 3 depicts the sample’s geometry established in the numerical model.

There are several FEM-based numerical modeling techniques that could be utilized to replicate the electromagnetic behavior of HTS, including the A-V formulation [20], the T-formulation [21], and the commonly used H-formulation [22], where the dependent variable is the magnetic field intensity and a nonlinear resistivity is utilized to analyze the electrical characteristics of the HTS material. Because of its excellent convergence, accuracy, reasonable computation time, and great agreement with experimental data, the H-formulation has been favored by most researchers across the world to simulate a range of HTS topologies [23,24]. In this investigation, we employ the well-studied FEA-based H-formulation model to carry out the numerical investigation.

Mathematically, the H-formulation utilizes the FEM to solve Faraday’s law:

(7) $$\nabla \times E=-\frac{\partial B}{\partial t}$$

Moreover, the quasi-static approximation of Ampere’s law yields the current density *J* from the magnetic field.

(8) ∇×H=J

To explain the unusual electrical properties of superconductors, we use the magnetic field *H* as a state variable and a nonlinear resistivity:

(9) $$\;\rho =\frac{E_{0}}{J}{\left(\frac{J}{J_{c}}\right)}^{n-1}$$

where *E*_0_ is the characteristic electric field, *J* is the current density, *J_c_* is the critical current density, and the value of *n* signifies the steepness of the transition from superconducting to normal state, commonly referred to as power factor. When *n* reaches infinity, the power law corresponds to the critical state model, also known as Bean’s critical state model [25]. Magnetically, the superconductor can be modelled as a material having relative magnetic permeability *μ_r_* = 1, Faraday’s equation in terms of the magnetic field takes the form:

(10) $$\frac{\partial \left(\mu_{0}\mu_{r}H\right)}{\partial t}+\nabla \times \left(\rho \nabla \times H\right)=0$$

The external magnetic fields could be introduced across the sample’s frontier in the air domain by carefully setting the Dirichlet boundary conditions. In addition, when *J* has a parallel component to *B*, the direction of the current density relative to the magnetic field influences the critical current density, resulting in force-free effects. Consequently, highly accurate *J_c_(B)* models with anisotropic dependence are required. Such anisotropic characteristics can be established by Kim’s model, as shown in Equation (7):

(11) $$J_{c}\left(B\right)=\frac{J_{c0}}{{\left(1+\frac{\left|B\right|}{B_{0}}\right)}^{m}}$$

Furthermore, for the calculation of AC loss, the power dissipation *E·J* is integrated over the domain of interest, as shown below:

(12) $$Q=\frac{2}{T}\int_{0.5T}^{T}\int_{Ω}^{}E\cdot J\;dΩdt\;$$

where *Ω* denotes the domain for computing AC loss and *T* denotes the duration of AC signal. Table 1 lists the other key parameters employed in the model.

## 4. Results and Discussion

**Figure 4:**
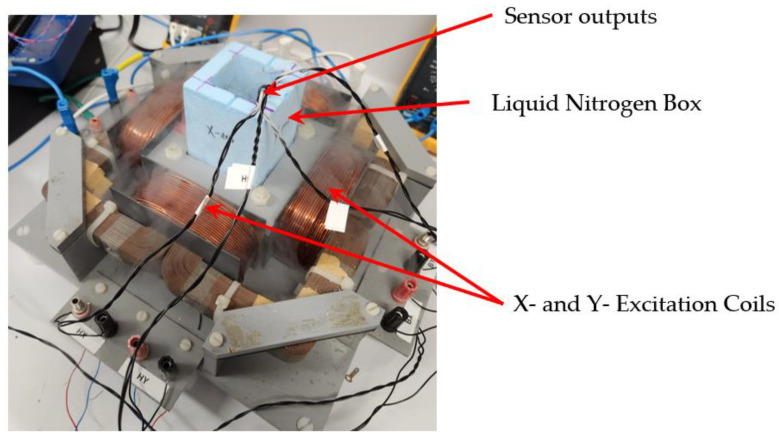
An image of the tester during experiment.

In this article, a new measurement approach recently reported by the authors in [18] has been extended to study the AC loss behavior in HTS bulks. A square HTS bulk specimen from Can Superconductors [26] with the dimensions of 40 mm × 40 mm × 10 mm was utilized, which was prepared by incorporating sensing coils for the sensing of components of B and H. To maintain the specimen in the superconducting area, it is carefully inserted in the specimen slot and immersed in liquid nitrogen. The level of liquid nitrogen is kept constant to maintain the desirable cryogenic condition required for superconductivity. Figure 4 depicts a view of the experimental setup, where gaseous nitrogen is plainly visible after expanding.

Once the experimental setup is deployed, and the cryogenic condition is sustained, the setup is organized for the measurements. When the specimen is subjected to an external field, the loss is absorbed instantly, resulting in an adiabatic state. In this experiment, the HTS sample is entirely immersed in liquid nitrogen, and the volume is continuously sustained in the container by frequently pouring the liquid nitrogen over the sample.

**Figure 5:**
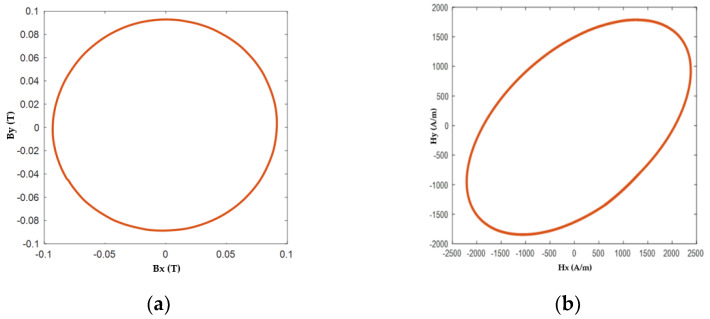
Loci of (**a**) Magnetic Flux Density (B) and (**b**) Magnetic Fields Strength (H) with 90 mT magnitude of B.

To study the AC loss characteristics in detail, the HTS specimen is subjected to various alternating and rotating magnetic flux density patterns with different amplitudes and frequencies as per the experimental methodology mentioned in Section 2. Furthermore, the results are also put in comparison with those from the numerical modeling methods. In order to confirm the rotation of the magnetic field in a purely circular form, Figure 5 depicts an example of the controlled loci of B and corresponding loci of H at 90 mT magnetic flux density and 50 Hz frequency. The loci of H are not circular when compared with the loci of B because the permeability differs with the value of B. Furthermore, the maximum values of Hx and Hy are not the same because the material responds differently when magnetized in X and Y directions.

**Figure 6:**
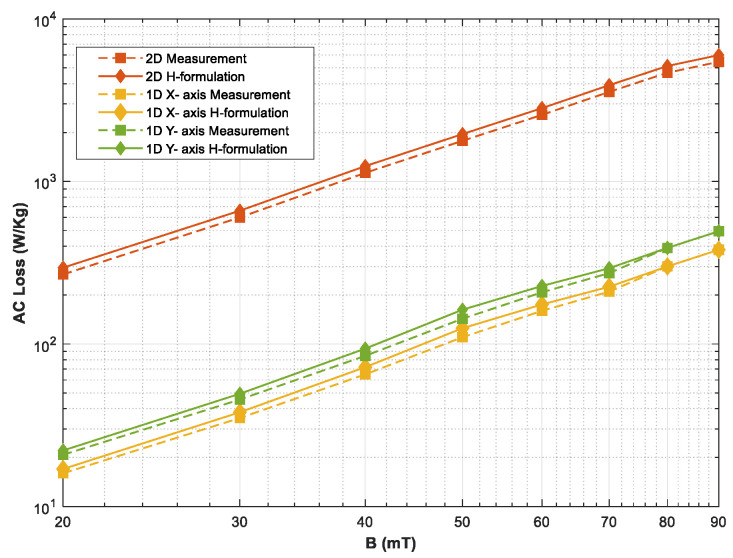
HTS AC loss variations at various alternating and rotating magnetic fields.

Figure 6 shows more comprehensive loss variations when the HTS sample is exposed to various alternating and rotating magnetic fields up to 90 mT. In order to compare the loss under rotating excitations, one-dimensional loss measurements are also obtained. In this case, only the coils in the particular orientations are excited to magnetize the sample in the X- or Y-axis, as described in Section 2. The loss under one-dimensional magnetic fields shows a traditional increment when the sample is exposed to higher fields. The subsequent results are also compared with the loss computed from numerical modeling, which shows similar behavior. Furthermore, the sample is exposed to various rotating magnetic fields in circular patterns, where both tester coils are excited as explained in Section 2. The results show that the AC power dissipative loss under rotating magnetic fields is significantly higher compared with the AC loss in one-dimensional AC loss, and it is linearly increasing with higher amplitudes of the magnetic fields. The two-dimensional experimental results also agree with the numerical investigations presented in earlier studies.

When the HTS bulks are considered in the rotating machines, this loss will add a significant burden on the cooling system, which will ultimately affect the cryogenic efficiency of the application. Therefore, the rotating loss must be considered while designing such large-scale applications. Moreover, the one-dimensional losses in X-axis and Y-axis differ from each other, which mainly relates to the traditional anisotropic behavior of the HTS material.

**Figure 7:**
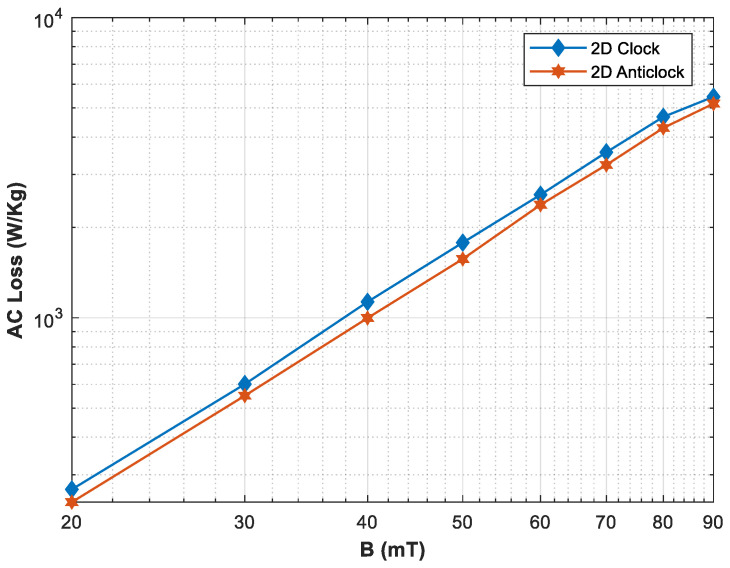
Comparison of measured AC loss subjected to rotating magnetic fields with clockwise and anticlockwise orientation.

**Figure 8:**
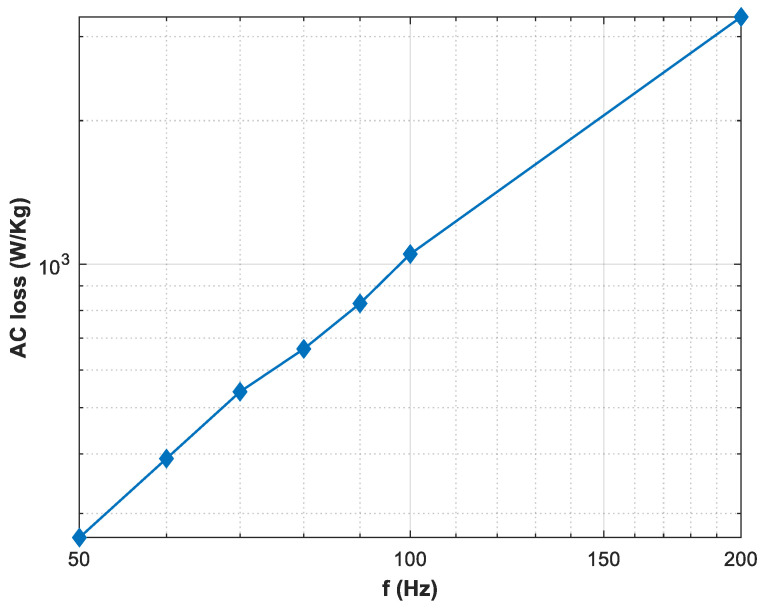
AC loss measured under rotating magnetic fields with multiple frequencies at 20 mT.

As the rotating magnetic field is a main phenomenon in rotating machines, the direction of the rotating magnetic field and the speed at which the magnetic field is rotating are also of significant importance when the HTS material needs to be characterized for rotating applications. Figure 7 shows the AC loss when the specimen is subjected to the rotating magnetic fields in clockwise and anticlockwise orientations. The increasing trend is similar in both of the orientations, but there is also a slight difference in the case, which relates to the anisotropic nature of the HTS material. Figure 8 shows the loss variation with the frequency of the rotating magnetic field. In this case, a 20 mT applied magnetic field rotates with a frequency of up to 200 Hz. The results show that AC power dissipation loss also increases significantly as the frequency of the applied magnetic fields is increased.

## 5. Conclusions

The measurement of AC loss is one of the most significant aspects of the HTS characterization. Most investigations concentrate on AC loss measurement and calculation in one-dimensional (1D) alternating magnetic fields created by either the transport current or by external magnetic excitations. However, when the HTS materials are incorporated into the rotating machines, they are exposed to the rotating magnetic fields. This exposure could complement to the AC loss, which will ultimately affect the cryogenic cooling efficiency of the HTS machines.

This study provides a detailed investigation of the AC loss measurement under various scenarios. The results are compared with those from finite element-based H-formulation. A square bulk HTS sample is used for this investigation which is submerged in the liquid nitrogen. The AC loss is obtained at circularly rotating flux density patterns of various amplitudes and is also compared with the AC loss under one-dimensional alternating flux density, where the rotating loss seems to be significantly higher. The loss variation has also been analyzed at various frequencies, where a significantly increasing trend is observed as the frequency is increased up to 200 Hz. The results presented in this article showcase further insights into the material characterization, which shows that the losses are actually higher in case of exposure under rotating magnetic fields. These results will be beneficial for the design of future large-scale HTS applications, where the additional power dissipation could be considered while planning the cryogenic cooling systems for such machines.

Moreover, this article also emphasizes the necessity for further research on the AC loss subjected to the rotating magnetic fields under various scenarios such as investigating various geometries of HTS and at even higher magnetic fields, as the loss is varied with the geometry of the sample, and the amplitude and orientation of the magnetic field.

## Figures and Tables

**Table 1:** Model Parameters.

Parameter	Symbol	Value
Power Factor	*n*	25
Critical Current Density	*J_c_*_0_	108 A·m^−2^
Characteristic Electric Field	*E*_0_	10^−4^ V m^−1^
Kim’s Model Arbitrary Parameter	*B*_0_	0.0041 T
Kim’s Model Arbitrary Parameter	*m*	0.5
Permeability of free space	*μ*_0_	4π × 10^−7^ H·m^−4^

## Data Availability

Not applicable.
